# Supplementary material for: Phenolic Profile and Bioactivity Changes of Lotus Seedpod and Litchi Pericarp Procyanidins: Effect of Probiotic Bacteria Biotransformation
Source: Antioxidants (Basel). 2023 Nov 7;12(11):1974. doi: 10.3390/antiox12111974 (PMC10669077; doi:10.3390/antiox12111974)
Supplement: Supplementary file 1 [file antioxidants-12-01974-s001.zip › antioxidants-2656216-supplementary.pdf]

Supporting Information

Phenolic Profile and Bioactivity Changes of Lotus Seedpod and Litchi Pericarp Procyanidins: Effect of Probiotic Bacteria Biotransformation

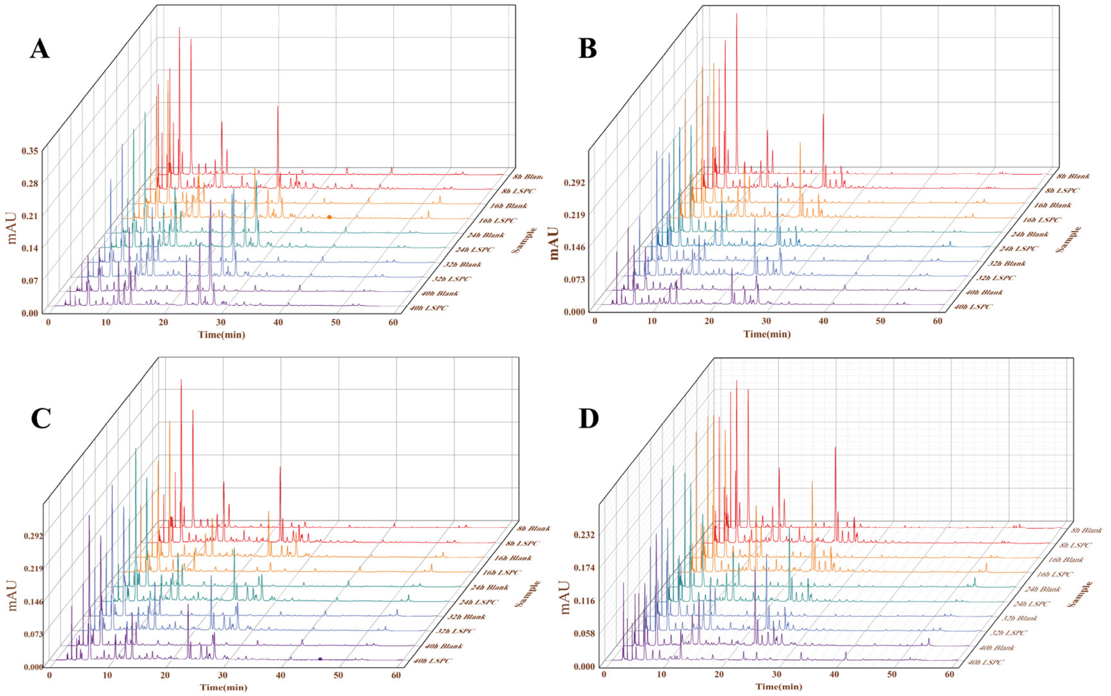

Figure. S1 Changes in procyanidin composition of LSPC metabolites during 48h incubated by *Lp90* (A), *ST81* (B), *HN001* (C) and *PP06* (D).

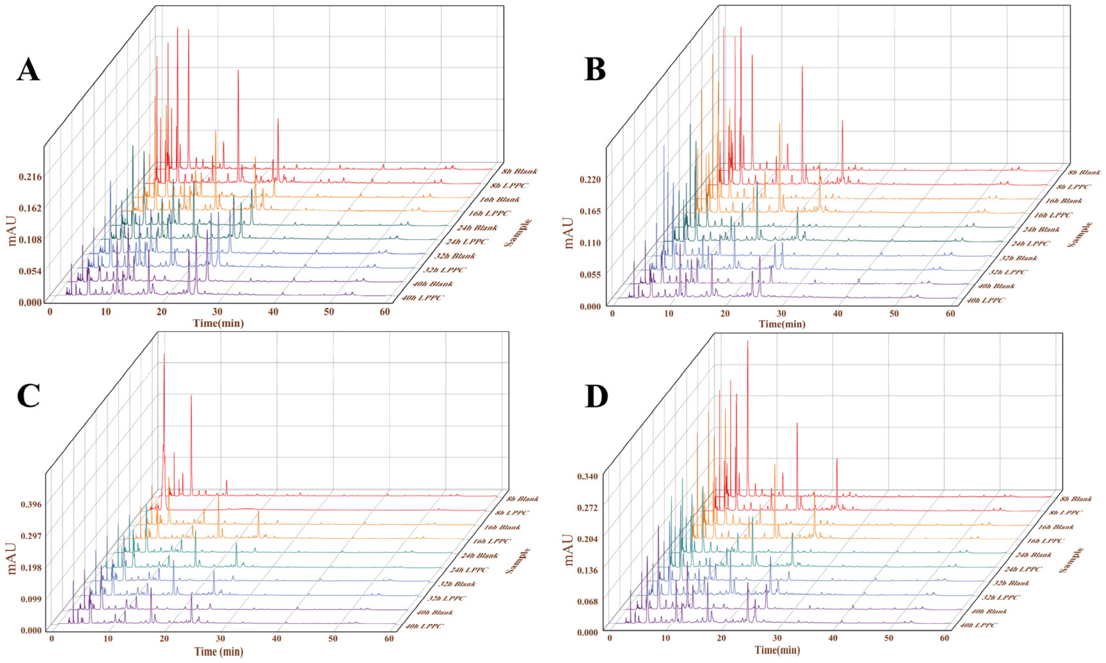

Figure. S2 Changes in procyanidin composition of LPPC metabolites during 48h incubated by *Lp90* (A), *ST81* (B), *HN001* (C) and *PP06* (D).

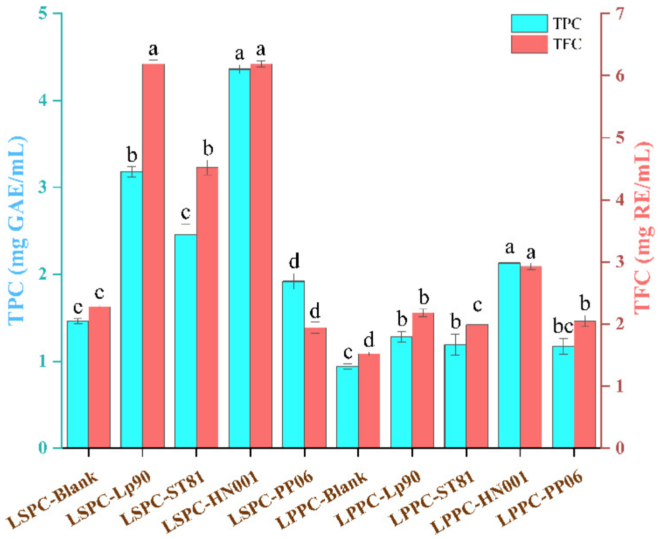

Figure S3 The TPC & TFC in the metabolites of LSPC and LPPC after 16 h of fermentation. Different lowercase letters indicate significant differences between metabolites from various LABs by the Tukey test ($p < 0.05$)

Table S1. Compounds identification, compound formula, retention times, measured m/z of molecular and mass fragments (MS/MS) in LSPC and LPPC respectively.

Sample	No.	Putative compounds	*RT min	m/z	MS/MS	Reference
LSPC	S1	Procyanidin B3	11.86	579.1492	450.0918, 287.0523	[1]
	S2	Catechin	13.11	291.0861	139.0426	[1]
	S3	Procyanidin B2	16.50	579.1492	291.0936	[1,2]
	S4	Rutin	22.71	610.0346	465.1029, 303.5012	[3]
	S5	Kaempferol 3-O-glucoside	24.96	449.1079	287.0511	[2,3]
	S6	Myricetin 3-O-glucoside	26.35	479.1208	479.1208, 317.0725	[1]
	S7	Syringetin 3-O-glucoside	26.52	509.1290	347.0823, 348.0814	[2]
LPPC	P1	Catechin	14.64	291.0725	13 9.0433	[1,4]
	P2	(-)-Epicatechin	16.47	291.0862	273.0375, 165.0191	[1,2]
	P3	A-type procyanidin trimer	17.14	865.1964	287.0529	[1,3]
	P4	Procyanidin A2	26.52	577.1335	287.0511	[2]

*Rt. retention

1. Lv, Q.; Luo, F.; Zhao, X.; Liu, Y.; Hu, G.; Sun, C.; Li, X.; Chen, K. Identification of proanthocyanidins from litchi (*Litchi chinensis Sonn.*) pulp by LC-ESI-Q-TOF-MS and their antioxidant activity. *PLoS One*. 2015, 10, e0120480. doi:10.1371/journal.pone.0120480.
2. Lyu, Q.; Kuo, T.H.; Sun, C.; Chen, K.; Hsu, C.C.; Li, X. Comprehensive structural characterization of phenolics in litchi pulp using tandem mass spectral molecular networking. *Food Chem*. 2019, 282, 9-17. doi:10.1016/j.foodchem.2019.01.001.
3. Xiao, J.S.; Xie, B.J.; Cao, Y.P.; Wu, H.; Sun, Z.D.; Xiao, D. Characterization of oligomeric procyanidins and identification of quercetin glucuronide from lotus (*Nelumbo nucifera Gaertn.*) Seedpod. *J Agr Food Chem*. 2012, 60, 2825-2829. doi:10.1021/jf205331e.
4. Zheng, Z.P.; Tan, H.Y.; Chen, J.; Wang, M. Characterization of tyrosinase inhibitors in the twigs of Cudrania tricuspidata and their structure-activity relationship study. *Fitoterapia*. 2013, 84, 242-247. doi:10.1016/j.fitote.2012.12.006.
